# PAM50 Breast Cancer Subtyping by RT-qPCR and Concordance with Standard Clinical Molecular Markers

**DOI:** 10.1186/1755-8794-5-44

**Published:** 2012-10-04

**Authors:** Roy RL Bastien, Álvaro Rodríguez-Lescure, Mark TW Ebbert, Aleix Prat, Blanca Munárriz, Leslie Rowe, Patricia Miller, Manuel Ruiz-Borrego, Daniel Anderson, Bradley Lyons, Isabel Álvarez, Tracy Dowell, David Wall, Miguel Ángel Seguí, Lee Barley, Kenneth M Boucher, Emilio Alba, Lisa Pappas, Carole A Davis, Ignacio Aranda, Christiane Fauron, Inge J Stijleman, José Palacios, Antonio Antón, Eva Carrasco, Rosalía Caballero, Matthew J Ellis, Torsten O Nielsen, Charles M Perou, Mark Astill, Philip S Bernard, Miguel Martín

**Affiliations:** 1 The ARUP Institute for Clinical and Experimental Pathology, Salt Lake City, UT, USA; 2 Department of Medical Oncology, Hospital Universitario de Elche, Elche, Spain; 3 Lineberger Comprehensive Cancer Center and Department of Genetics and Department of Pathology & Laboratory Medicine, University of North Carolina at Chapel Hill, Chapel Hill, NC, USA; 4 Department of Medicine, Universitat Autónoma de Barcelona, Barcelona, Spain; 5 Department of Medical Oncology, Hospital Universitario La Fe, Valencia, Spain; 6 Department of Medical Oncology, Hospital Universitario Virgen del Rocío, Sevilla, Spain; 7 Department of Medical Oncology, Hospital de Donostia, San Sebastián, Spain; 8 Department of Medical Oncology, Corporatiò Sanitaria Parc Taulí, Sabadell, Spain; 9 Department of Oncological Sciences, Huntsman Cancer Institute, Salt Lake City, UT, USA; 10 Department of Medical Oncology, Hospital Universitario Virgen de la Victoria, Málaga, Spain; 11 Department of Pathology, University of Utah Health Sciences Center/Huntsman Cancer Institute, Salt Lake City, UT, USA; 12 Department of Pathology, Hospital General Universitario de Alicante, Alicante, Spain; 13 Department of Pathology, Hospital Virgen del Rocio, Sevilla, Spain; 14 Department of Medical Oncology, Hospital Universitario Miguel Servet, Zaragoza, Spain; 15 Spanish Breast Cancer Research Group, GEICAM, Madrid, Spain; 16 Department of Oncology, Washington University, St. Louis, MO, USA; 17 Department of Anatomical Pathology, University of British Columbia, Vancouver, Canada; 18 Department of Medical Oncology, Hospital General Universitario Gregorio Marañón, Universidad Complutense, Madrid, Spain; 19 Huntsman Cancer Institute, 2000 Circle of Hope, Salt Lake City, UT, 84112, USA

## Abstract

**Background:**

Many methodologies have been used in research to identify the “intrinsic” subtypes of breast cancer commonly known as Luminal A, Luminal B, HER2-Enriched (HER2-E) and Basal-like. The PAM50 gene set is often used for gene expression-based subtyping; however, surrogate subtyping using panels of immunohistochemical (IHC) markers are still widely used clinically. Discrepancies between these methods may lead to different treatment decisions.

**Methods:**

We used the PAM50 RT-qPCR assay to expression profile 814 tumors from the GEICAM/9906 phase III clinical trial that enrolled women with locally advanced primary invasive breast cancer. All samples were scored at a single site by IHC for estrogen receptor (ER), progesterone receptor (PR), and Her2/neu (HER2) protein expression. Equivocal HER2 cases were confirmed by chromogenic in situ hybridization (CISH). Single gene scores by IHC/CISH were compared with RT-qPCR continuous gene expression values and “intrinsic” subtype assignment by the PAM50. High, medium, and low expression for *ESR1*, *PGR*, *ERBB2*, and proliferation were selected using quartile cut-points from the continuous RT-qPCR data across the PAM50 subtype assignments.

**Results:**

*ESR1*, *PGR*, and *ERBB2* gene expression had high agreement with established binary IHC cut-points (area under the curve (AUC) ≥ 0.9). Estrogen receptor positivity by IHC was strongly associated with Luminal (A and B) subtypes (92%), but only 75% of ER negative tumors were classified into the HER2-E and Basal-like subtypes. Luminal A tumors more frequently expressed PR than Luminal B (94% vs 74%) and Luminal A tumors were less likely to have high proliferation (11% vs 77%). Seventy-seven percent (30/39) of ER-/HER2+ tumors by IHC were classified as the HER2-E subtype. Triple negative tumors were mainly comprised of Basal-like (57%) and HER2-E (30%) subtypes. Single gene scoring for *ESR1*, *PGR*, and *ERBB2* was more prognostic than the corresponding IHC markers as shown in a multivariate analysis.

**Conclusions:**

The standard immunohistochemical panel for breast cancer (ER, PR, and HER2) does not adequately identify the PAM50 gene expression subtypes. Although there is high agreement between biomarker scoring by protein immunohistochemistry and gene expression, the gene expression determinations for *ESR1* and *ERBB2* status was more prognostic.

## Background

For over a decade, research studies have used gene expression to classify invasive breast cancers into biologically and clinically distinct subtypes that have become known as Luminal A, Luminal B, HER2-Enriched (HER2-E) and Basal-like [1-3]. Subtype information has repeatedly shown to be an independent predictor of survival in breast cancer when used in multivariate analyses with standard clinical-pathological variables [3-6]. In 2009, Parker et al. derived a minimal gene set (PAM50) for classifying “intrinsic” subtypes of breast cancer [3,7]. The PAM50 gene set has high agreement in classification with larger “intrinsic” gene sets previously used for subtyping [1,3,4,8], and is now commonly employed [9-12].

There are several multi-gene expression tests clinically available for determining risk of relapse in early stage breast cancer, including the 21-gene recurrence score [13] (Oncotype Dx®, Genomic Health Inc, Redwood City, CA, http://www.oncotypedx.com), the 14-gene distant metastasis signature [14] (BreastOncPx™, US Labs, Irvine, CA, http://www.uslabs.net), the 97-gene histologic grade predictor [15] (MapQuant Dx™ Genomic Grade, Ipsogen, Marseilles, France and New Haven, CT, USA, http://www.ipsogen.com), and the 70-gene prognosis signature [16] (MammaPrint®, Agendia, Irvine, CA, http://www.agendia.com). The molecular signature of proliferation is perhaps the strongest variable in all these tests for determining outcome in ER + breast cancer.

In addition to gene expression profiling by microarray or RT-qPCR [2-4,8,17,18], many studies have used immunohistochemical panels to identify subtypes [19-21]. For example, high grade ER+/HER2- tumors and ER+/HER2+ tumors are often considered Luminal B, while ER-/HER2+ are considered HER2-E subtype and triple negative tumors are considered Basal-like. In this study, we assess agreement between histopathology/IHC status and PAM50 classification for subtype, *ESR1*, *PGR*, *ERBB2*, and proliferation.

## Methods

### Samples and clinical data

There was ethical review and approval for all protocols used in this study from the respective centers involved and all subjects gave written informed consent to participate. A training set was developed using 171 breast samples, comprised of 16 “normal” breast tissue samples from reduction mammoplasties or grossly uninvolved breast tissue and 155 primary invasive breast cancers. These samples were collected from 2005–2009 under IRB approved protocols at the University of Utah and the University of North Carolina at Chapel Hill. Clinical-pathological information associated with the samples is based on the College of American Pathology (CAP) and American Joint Committee on Cancer (AJCC) standards at the time of collection (Additional file 1). Subtype classification and single and meta-gene (proliferation) scores were predicted on an independent test set of 814 samples from the GEICAM/9906 clinical trial, a randomized Phase 3 trial of fluorouracil, epirubicin, and cyclophosphamide alone or followed by paclitaxel [22]. Patients that were hormone receptor positive (ER and/or PR positive by IHC) were given adjuvant tamoxifen. The hormone receptor status for these samples was evaluated at a single site (Department of Pathology, Hospital General Universitario de Alicante) using immunohistochemistry (IHC) for progesterone receptor (PR) (clone PgR636, DAKO, Glostrup, Denmark) and estrogen receptor (ER) (clone 1D5, DAKO, Glostrup, Denmark) (Additional file 2). The scores for the proportion of dyed cells and intensity were summed to obtain a total Allred Score [23]. Measurement of HER2 expression was performed by Herceptest™ (DAKO, Glostrup, Denmark) and samples with scores of 2+ by IHC were confirmed by CISH, following the ASCO/CAP guidelines [24]. The clinical data for the training set and GEICAM/9906 test set are summarized in Table 1. 

**Table 1:** Patient characteristics

**Variable**	**Training set data**	**n = 154 Total (%)**	**Variable**	**Test set data**	**n = 814 Total (%)**
Age (years)	Median	55.5	Age (years)	Median	50.4
	(range)	26 – >92		(range)	23.1 – 76.2
Menopausal status	Pre	49 (31.8)	Menopausal status	Pre	439 (53.9)
	Post	101 (65.6)		Post	375 (46.1)
	Unknown	4 (2.6)			
Primary tumor size	T1	63 (40.9)	Primary tumor size	T1	338 (41.5)
	T2	69 (44.8)		T2	430 (52.8)
	T3	17 (11.0)		T3	46 (5.7)
	Unknown	1 (0.6)			
	Reduction Mamoplasty	4 (2.6)			
Nodal status	0	95 (61.7)	Nodal status	0	0
	1 – 3	54 (35.1)		1 – 3	503 (61.8)
	> 3	0 (0)		> 3	311 (38.2)
	Unknown	1 (0.6)			
	Reduction Mamoplasty	4 (2.6)			
Histopathologic grade*	G1	23 (14.9)	Histopathologic grade*	G1	107 (13.1)
	G2	45 (29.2)		G2	335 (41.2)
	G3	80 (51.9)		G3	313 (38.5)
	GX	2 (1.3)		GX	59 (7.2)
	Reduction Mamoplasty	4 (2.6)			
Estrogen receptor^	Positive	100 (64.9)	Estrogen receptor^	Positive	644 (79.1)
	Negative	49 (31.8)		Negative	170 (20.9)
	Unknown	1 (0.6)			
	Reduction Mamoplasty	4 (2.6)			
Progesterone receptor^^	Positive	82 (53.2)	Progesterone receptor^^	Positive	567 (69.7)
	Negative	67 (43.5)		Negative	247 (30.3)
	Unknown	1 (0.6)			
	Reduction Mamoplasty	4 (2.6)			
HER2 status	Positive	37 (24.0)	HER2 status	Positive	116 (14.3)
	Negative	111 (72.1)		Negative	698 (85.7)
	Unknown	2 (1.3)			
	Reduction Mamoplasty	4 (2.6)			
Ki67 IHC	Unknown	154 (100)	Ki67 IHC	Positive	236 (29.6)
				Negative	561 (70.4)
PAM50 Intrinsic Subtype	Luminal A	53 (34.4)	PAM50 Intrinsic Subtype	Luminal A	277 (34.0)
	Luminal B	27 (17.5)		Luminal B	261 (32.1)
	HER2-enriched	32 (20.8)		HER2-enriched	174 (21.4)
	Basal-like	38 (24.7)		Basal-like	70 (8.6)
	Normal-like	4 (2.6)		Normal-like	32 (3.9)

*Grade based on Nottingham-Bloom-Richardson scoring.

^ER positive required at least 10% staining nuclei.

^^PR positive required at least 10% staining nuclei.

^#^HER2 positive were 3+ IHC or 2+ and CISH confirmed.

### Measurement of PCR efficiency, limits of detection, and limits of quantification

Breast cancer cell lines (BT474, MCF7, MDA-MB-231, MDA-MB-436, MDA-MB-453, MDA-MB-468, SKBR3 and T47D) were cultured, pelleted and processed into FFPE tissue blocks. The RNA was extracted, pooled, reverse transcribed, and serially diluted at 2-fold increments from 2.56μg to 0.039ng per assay, which corresponds to a range of 7.11ng to 108.51fg of cDNA per reaction well. Each gene was measured in triplicate per RT-qPCR run on the Roche LC480 and 2 runs were performed for each of the 17 dilutions. A detailed description of methods used to calculate PCR efficiency, limits of detection and limits of quantification can be found in Additional file 3.

### Selection of prototype samples for the RT-qPCR training set

Training set samples were run across 3 batches of PCR plates manufactured at ARUP Laboratories (ARUP Laboratories, Salt Lake City, UT, http://www.aruplab.com). The method to identify prototype samples representing the subtypes has been previously described [3]. Briefly, hierarchical clustering (median centered by gene, Pearson correlation, centroid-linkage) [25] was performed on the RT-qPCR data and SigClust was run at each node of the dendrogram beginning at the root and stopping when the test was no longer significant (p > 0.001). A “centroid” was generated for each subtype in the training set using the average expression for each gene across all prototype samples of a given subtype. Single sample subtype prediction was performed by calculating a Spearman rank correlation coefficient between the gene expression values of an individual sample compared to each of the centroid gene values for Luminal A, Luminal B, HER2-Enriched, Basal-like, and Normal. The subtype classification for the new sample is assigned to the centroid with the highest correlation.

### 10-Fold cross validation to determine stability of selected prototypes

The 154 prototype samples identified by SigClust were randomly split into 10 groups. Nine of the 10 groups were used to calculate new centroids for each of the 5 possible subtype assignments. Each sample from the remaining group was then assigned a subtype based on closest proximity to the newly calculated centroids using Spearman's Rho. The process of calculating centroids using 9 of the 10 groups and predicting on the remaining group was repeated leaving out a different group each time.

### Measurements of assay reproducibility

Reproducibility of the PAM50 assay was determined using 3 cell lines (MCF7, ME16C and SKBR3) and a pool of Luminal A prototype samples that were each run 12 times (3 runs across 4 batches of PAM50 plates) over 30 days. Variation in each gene measurement was assessed using the difference between the mean calibrator crossing point (C_P_) and each sample replicate C_P_ (ΔC_P_). The square root of the mean CV^2^ for ΔC_P_ was used to estimate the variation for each gene within plate, within batch, and across batches. Higher gene CVs may be due to lower concentration of a single gene within a sample. We used the technical variability in measuring each gene to further assess the stability of the categorical subtype call in the GEICAM/9906 test set samples. Since the biology between subtypes is a continuum and some samples may have close proximity to more than 1 prototypic subtype, we used a Monte-Carlo simulation to introduce random error into the call to determine the frequency of switching subtype [26].

### Scaling single and Meta-Gene scores

The PAM50 subtype assay can also provide quantitative and qualitative gene expression scores for the standard biomarkers usually measured semi-quantitatively by IHC: *ESR1*/ER, *PGR*/PR and *ERBB2*/HER2. In addition, the PAM50 contains many cell cycle regulated genes that can be combined into a meta-gene for proliferation (*CENPF, ANLN, CDC20, CCNB1, CEP55, MYBL2, MKI67, UBE2C, RRM2,* and *KIF2C*). The meta-gene for proliferation were selected because they had strong correlation within the associated dendrogram of the training set cluster. The quantitative scale of 1–10 for the single genes and proliferation was derived by rescaling the original log-expression ratios from the training set and included a 10% buffer on either side of the original values to allow for values that were higher or lower than what was encountered in the training set. Any new values that were less than 0 or greater than 10 were truncated at 0 and 10, respectively.

Fixed cut-points (low vs. intermediate/high) for the single genes (*ESR1*, *PGR*, and *ERBB2*) and proliferation were directly applied from the training set to the GEICAM/9906 test set. Receiver Operator Characteristic (ROC) curves were generated by dichotomizing IHC data and treating RT-qPCR data as a continuous variable.

## Results

### Training set, subtype stability, and classification accuracy

**Figure 1:**
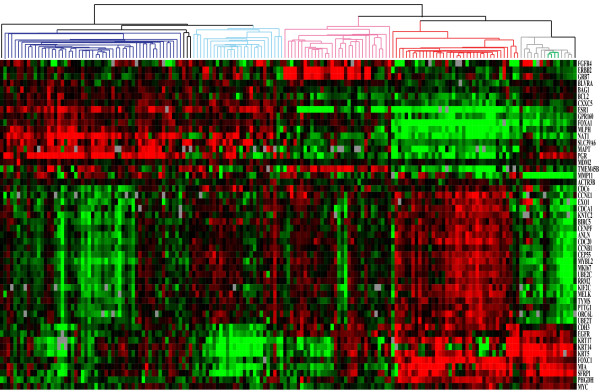
**Clinical PAM50 RT-qPCR breast cancer training set.** Hierarchical clustering of RT-qPCR data for the PAM50 classifier genes normalized to the 5 control genes using 171 FFPE procured breast samples. Statistical selection using SigClust identified the 5 significant groups previously identified and designated as Luminal **A** (dark blue), Luminal **B** (light blue), HER2-E (pink), Basal-like (red), and Normal (green). The 16 non-neoplastic samples (grey), from reduction mammoplasty and grossly uninvolved breast tissues, all Clustered together and away from the invasive cancers. SigClust identified 4 reduction mammoplasty samples (green) that were used to train the Normal subtype.

We identified 154 prototypic samples from the RT-qPCR data by hierarchical clustering of the PAM50 classifier genes, and statistical selection from the dendrogram by SigClust [27]. The training set was comprised of 53 Luminal A, 27 Luminal B, 32 HER2-enriched, 38 Basal-like and 4 Normal-like (Figure 1). The 10-fold cross validation had 91.6% concordance (multi-rater kappa score of 0.885) with the initial SigClust subtype assignments (Additional file 4). 

### Interference from normal breast tissue contamination

A major source of subtype misclassification comes from having normal tissue within the tumor sample [28]. We assessed the effect of having contaminating normal tissue within the tumor sample by diluting out RNA from tumor subtypes with pooled RNA from “normal” reduction mammoplasties (0%, 25%, 50% and 75%). Primary tumors were pooled to represent Luminal A and HER2-E samples while cell lines were used to represent Luminal B (MCF7) and Basal-like (ME16C). The changes in subtype classification occurred in a systematic fashion with all subtypes switching directly to a classification of Normal, with the exception of Luminal B, which switched to Luminal A. The switch from Luminal B to Luminal A required 50% contribution from the normal breast tissue signature. Interference data from the introduction of normal breast tissue RNA into each of the subtypes is provided in Additional file 5. During the dilution series for HER2-E with “normal” there was switching in the *ESR1* score between intermediate and low suggesting that both samples had similar *ESR1* expression near the cut-off for those scores.

### Subtype, immunohistochemistry, and RT-qPCR gene scores

**Figure 2:**
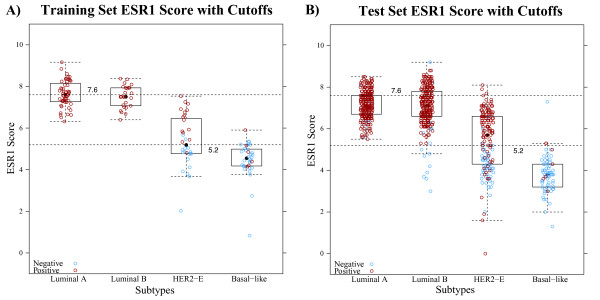
***ESR1* score cut-offs using training set and the GEICAM/9906 testing set.** The *ESR1* score is provided as a qualitative call of high, intermediate, or low. The cut-offs were based on the continuous expression of *ESR1* across prototype samples in the training set. Each circle on the box plot represents an individual sample that is color coded according to IHC status. The cut-points between high, intermediate, and low classes were individually derived from the training set samples (**A**). Data from *ESR1* gene expression over the GEICAM 9906 samples (**B)** are plotted on the same scale as the training set. Samples are colored according to ER IHC positivity (red) or negativity (blue) determined at a central facility.

**Figure 3:**
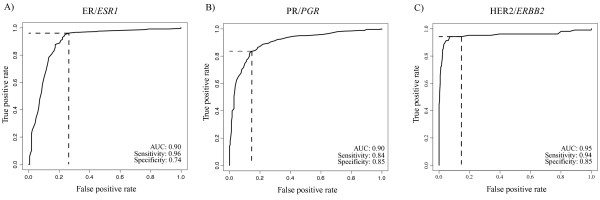
**Receiver Operator Characteristic (ROC) curves for* ESR1*,* PGR*, and *ERBB2* for the GEICAM/9906 test set.** ROC curves for the GEICAM/9906 test set were generated using the clinical IHC status (positive vs. negative) for ER, PR, and HER2/*neu* as compared to the continuous RT-qPCR data for *ESR1*, *PGR*, and *ERBB2.* The cut-points for sensitivity/specificity are based on the training set.

The RT-qPCR values for *ESR1*, *PGR*, *ERBB2*, and proliferation were evaluated across prototypic samples in the training set. High, intermediate, and low cut points were made based on the continuous distribution of expression across the tumor subtypes. The cut-points for each of the scores and how they were determined is presented in Table 2. Figure 2 shows the expression and cut-points for *ESR1* in the training set and how these compare within the GEICAM/9906 test set. Additional single and meta-gene cut-points for the training and test sets can be found in Additional file 6. Comparisons between the gene expression and IHC data for GEICAM/9906 gave good overall agreement with a high area under the curve (AUC) for *ESR1*/ER (AUC = 0.90), *PGR*/PR (AUC = 0.90), and *ERBB2*/HER2 (AUC = 0.95) (Figure 3). Rather than re-optimize the cut-points on the test set, the fixed cut-points based on the training set were used and it showed high sensitivity/specificity, although a slightly higher false positive rate for *ERBB2* than would have been selected by eye.

**Table 2:** Cut-points for quantitative gene scores

	**Score ranges**		
**Genes/Meta-genes**	**Low**	**Intermediate**	**High**
*ESR1* (ER)^†^	0 - 5.2	>5.2 - 7.6	>7.6 - 10
*PGR* (PR)^‡^	0 - 5.1	>5.1 - 7.4	>7.4 - 10
*ERBB2* (HER2)^§^	0 - 5.6	>5.6 - 7.5	>7.5 - 10
Proliferation^*^	0 - 3.9	>3.9 - 5.3	>5.3 - 10

^†^high *ESR1* = above median expression for Luminal A; low *ESR1* = below median expression for HER2-enriched.

^‡^high *PGR* = above median expression for Luminal A; low *PGR* = below median expression for Luminal B.

^§^high *ERBB2* = above median expression for HER2-enriched; low *ERBB2* = below lowest quartile expression for HER2-enriched.

^*^high Proliferation = above highest quartile expression for Luminal A; low Proliferation = below lowest quartile expression for Luminal A.

Ninety-two percent (497/538) of Luminal (A/B) tumors were ER + by IHC and 99% (530/538) had an intermediate-high *ESR1* score (Tables 3 and 4). Luminal A tumors more frequently expressed PR/*PGR* than Luminal B tumors using either IHC (94% vs 74%) or qPCR (95% vs 61%).

**Table 3:** Histological scoring across PAM50 subtypes

	**Grade**	**ER**	**PR**	**HER2**
** LumA**	G1-68 (25%)	Neg-19 (7%)	Neg-16 (6%)	Neg-273 (99%)
**n = 277**	G2-142 (51%)	Pos-258 (93%)	Pos-261 (94%)	Pos-4 (1%)
	G3-39 (14%)			
	GX-28 (10%)			
** LumB**	G1-25 (10%)	Neg-22 (8%)	Neg-68 (26%)	Neg-224 (86%)
**n = 261**	G2-111 (43%)	Pos-239 (92%)	Pos-193 (74%)	Pos-37 (14%)
	G3-111 (43%)			
	GX-14 (5%)			
**HER2-E**	G1-6 (3%)	Neg-63 (36%)	Neg-93 (53%)	Neg-105 (60%)
**n = 174**	G2-65 (37%)	Pos-111 (64%)	Pos-81 (47%)	Pos-69 (40%)
	G3-96 (55%)			
	GX-7 (4%)			
** Basal**	G1-0 (0%)	Neg-63 (90%)	Neg-62 (89%)	Neg-67 (96%)
**n = 70**	G2-4 (6%)	Pos-7 (10%)	Pos-8 (11%)	Pos-3 (4%)
	G3-61 (87%)			
	GX-5 (7%)			

**Table 4:** Single gene scores and proliferation across PAM50 subtypes

	**Proliferation**	**ESR1^**	**PGR^**	**ERBB2^**
**LumA**	Low-104 (38%)	Neg-0 (0%)	Neg-15 (5%)	Neg-254 (92%)
**n = 277**	Intermediate-116 (42%)	Pos-277 (100%)	Pos-262 (95%)	Pos-23 (8%)
	High-57 (11%)			
**LumB**	Low-4 (2%)	Neg-8 (3%)	Neg-101 (39%)	Neg-194 (74%)
**n = 261**	Intermediate-55 (21%)	Pos-253 (97%)	Pos-160 (61%)	Pos-67 (26%)
	High-202 (77%)			
**HER2-E**	Low-15 (9%)	Neg-76 (44%)	Neg-112 (64%)	Neg-81 (47%)
**n = 174**	Intermediate-60 (34%)	Pos-98 (56%)	Pos-62 (36%)	Pos-93 (53%)
	High-99 (57%)			
**Basal**	Low-0 (0%)	Neg-67 (96%)	Neg-67 (96%)	Neg-66 (94%)
**n = 70**	Intermediate-3 (4%)	Pos-3 (4%)	Pos-3 (4%)	Pos-4 (6%)
	High-67 (96%)			

^ RT-qPCR status based on intermediate-high (positive) versus low (negative).

**Figure 4:**
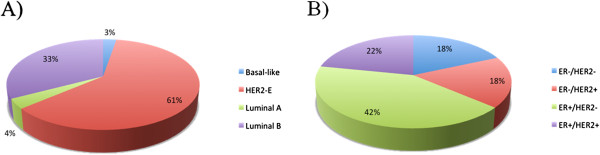
**Association between HER2 status and “intrinsic” subtype.** Figure (**A**) shows the subtype distribution within HER2+ samples by IHC/CISH. Figure (**B**) shows the ER/HER2 status for samples only within the HER2-E subtype.

Although the HER2-E subtype is often thought of as being ER-, only 36% (63/174) were ER- by IHC and 44% (76/174) were low *ESR1* score. Seventeen percent of HER2-E samples were called triple-negative. Of the clinically HER2+ group by IHC/CISH, approximately two-thirds (69/113 = 61%) were HER2-E and one-third were Luminal B (37/113 = 33%) subtype (Figure 4). Using the qPCR cut-off for *ERBB2* expression, we found that 98% (609/624) of samples that were low *ERBB2* were also HER2- by IHC/CISH, while 53% (109/190) of tumors with intermediate-high *ERBB2* expression were HER2+. However, analyses just within the HER2-E subtype showed 71% (66/93) of tumors with high *ERBB2* gene expression were HER2+ by IHC/CISH.

**Figure 5:**
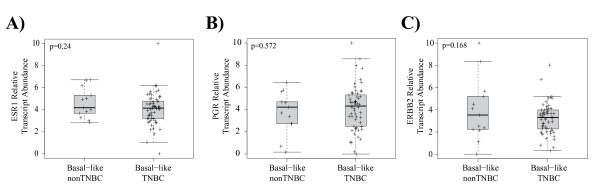
**Relative transcript abundance for *ESR1*,* PGR*, and* ERBB2* in the Basal-like subtype.** There was no difference (p > 0.05) in (**A**) *ESR1*, (**B**) *PGR*, or (**C**) *ERBB2* expression by qPCR in Basal-like tumors, regardless of being called triple-negative or non-triple negative by IHC/CISH.

Ninety percent (63/70) of Basal-like tumors were ER- by IHC and 96% (67/70) were low *ESR1* score. Furthermore, 81% (57/70) of Basal-like tumors were triple negative (ER-/PR-/HER2-) by IHC/CISH and 86% (60/70) were low in all 3 genes by qPCR (Table 5). Conversely, only 56% (57/101) and 67% (60/90) of triple negatives defined by IHC/CISH or qPCR were Basal-like, respectively. There was no difference (p > 0.05) in *ESR1*, *PGR* or *ERBB2* expression by qPCR in Basal-like tumors, regardless of being triple-negative or non-triple negative by IHC/CISH (Figure 5).

**Table 5:** Surrogate subtyping by 3-marker scoring

	**Luminal A**	**Luminal B**	**HER2-E**	**Basal-like**
ER+/PR+/HER2- (n = 471)	244 (52%)	170 (36%)	53 (11%)	4 (1%)
*ESR1*+/*PGR*+/*ERBB2*- (n = 397)	239 (60%)	127 (32%)	31 (8%)	0 (0%)
ER+/PR-/HER2- (n = 81)	12 (15%)	47 (58%)	19 (23%)	3 (4%)
*ESR1*+/*PGR-*/*ERBB2*- (n = 101)	15 (15%)	65 (64%)	18 (18%)	3 (3%)
ER-/PR-/HER2+ (n = 39)	0 (0%)	7 (18%)	30 (77%)	2 (5%)
*ESR1-*/*PGR-*/*ERBB2*+ (n = 51)	0 (0%)	6 (12%)	41 (80%)	4 (8%)
ER-/PR-/HER2- (n = 101)	4 (4%)	10 (10%)	30 (30%)	57 (56%)
*ESR1-*/*PGR-*/*ERBB2*- (n = 90)	0 (0%)	2 (2%)	28 (31%)	60 (67%)
ER+/PR+/HER2+ (n = 45)	2 (4%)	18 (40%)	25 (56%)	0 (0%)
*ESR1*+/*PGR*+/*ERBB2*+ (n = 80)	23 (29%)	33 (41%)	24 (30%	0 (0%)
ER+/PR-/HER2+ (n = 18)	0 (0%)	4 (22%)	14 (78%)	0 (0%)
*ESR1*+/*PGR-*/*ERBB2*+ (n = 53)	0 (0%)	28 (53%)	25 (47%)	0 (0%)
ER-/PR+/HER2- (n = 25)	14 (56%)	4 (16%)	3 (12%)	4 (16%)
*ESR1-*/*PGR*+/*ERBB2*- (n = 7)	0 (0%)	0 (0%)	4 (57%)	3 (43%)
ER-/PR+/HER2+ (n = 2)	1 (50%)	1 (50%)	0 (0%)	0 (0%)
*ESR1-*/*PGR*+/*ERBB2*+ (n = 3)	0 (0%)	0 (0%)	3 (100%)	0 (0%)

Additional file 7 shows a comparison of unsupervised hierarchical clustering with supervised subtype assignment and single marker scores for GEICAM/9906. In general, the supervised classification agreed with the sample associated dendrogram clusters. The side branches of the dendrogram clusters are less correlated to other samples and reflect the continuum in the biology, especially between Luminal A, Luminal B and HER2-E subtypes. The HER2-E and Basal-like subtypes cluster away from the Luminal tumors and have similar gene expression profiles overall; however, standard IHC/CISH biomarkers poorly define these subtypes.

### Prognostic significance of gene expression versus standard methods for ER and HER2 status

**Figure 6:**
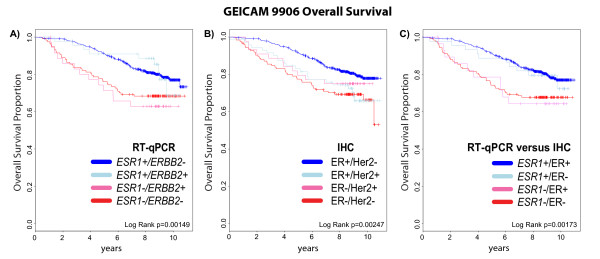
**Kaplan-Meier plots of overall survival in GEICAM 9906 data set.** When stratifying all patients with locally advanced breast cancer, regardless of chemotherapy regimen (FEC vs FEC-T), both RT-qPCR (**A**) and IHC/CISH (**B**) molecular classifications for assessing *ESR1*/ER and *ERBB2/*Her2 status were significant. However, the separation of the survival curves suggests that ER-status as assessed by qPCR has prognostic superiority to IHC (**C**).

Although there was high agreement between IHC/CISH and RT-qPCR measurements for ER/*ESR1* and HER2/*ERBB2*, we wanted to assess whether the two different methods provided equivalent prognostic information. When tested in a multivariate Cox model for overall survival, only the RT-qPCR assignments were selected in the final Cox model in the GEICAM/9906 test set (Table 6). When all patients with locally advanced breast cancer were stratified, regardless of chemotherapy regimen (FEC vs FEC-T), both classifications for assessing ER/*ESR1* and HER2/*ERBB2* status were significantly associated with outcome (Figure 6). Since endocrine therapy was based on ER status determined by IHC, those ER + samples that were *ESR1*- (29/154 = 19%) would have received adjuvant tamoxifen and conversely those patients with ER- tumors that were *ESR1*+ (45/660 =7%) would not have received therapy. When separating outcome based on agreement and disagreement between the methods, we find that women with ER+/*ESR*1+ tumors have similar outcomes to women with ER-/*ESR1*+ tumors, and women with ER-/*ESR*1- tumors have similar outcomes to women with ER+/*ESR1*- tumors. This shows that the RT-qPCR assignment is more prognostic and accurate than IHC for ER.

**Table 6:** Univariate and multivariate analyses of prognostic factors in GEICAM/9906

**MVA analysis for OS**			**Univariate analysis**			**Multivariate analysis (backward/forward stepwise selection)**		
**Signatures**	**HR**	**Lower 95%**	**Upper 95%**	**p-value**	**HR**	**Lower 95%**	**Upper 95%**	**p-value**
ARM FEC-P vs. FEC	0.708	0.528	0.948	0.021	0.734	0.543	0.993	0.045
Grade 3 vs. 1-2	1.745	1.297	2.346	<0.001	1.335	0.962	1.853	0.084
Nodes >3 vs. 1-3	2.103	1.574	2.808	<0.001	1.882	1.391	2.546	0.000
Tumor size >2 cm vs. ≤2cm	2.089	1.510	2.890	<0.001	1.724	1.224	2.427	0.002
Age >50 vs ≤50	1.189	0.890	1.589	0.242	1.012	0.999	1.026	0.078
ER status by IHC + vs. -	0.619	0.449	0.855	0.004	-	-	-	-
ER status by GE + vs. -	0.536	0.387	0.741	<0.001	0.630	0.438	0.906	0.013
Clinical HER2 status + vs. -	1.389	0.9532	2.024	0.0872	-	-	-	-

## Discussion

Many studies have tried to identify the gene expression-based “intrinsic” subtypes using a variety of methods for the sake of simplicity, cost, and available technologies. Methods that can be used from formalin-fixed, paraffin-embedded tissues are optimal since this is how samples are procured and archived in most pathology departments. The two preferred technologies for gene expression profiling from FFPE tissues are RT-qPCR [17,18] and Nanostring nCounter [29]. The nCounter system uses color-coded probes that bind directly to the RNA transcript without reverse transcription and PCR amplification. While these methods have high agreement for gene quantification, other methodologies may lead to different conclusions and treatment decisions. For instance, in the NCIC.CTG MA.12 clinical trial that randomized pre-menopausal women with primary breast cancer to tamoxifen versus placebo it was found that a panel 6 IHC antibodies for subtyping was not prognostic but the PAM50 RT-qPCR subtypes were prognostic [30]. In another randomized study (NCIC.CTG MA.5) that assessed PAM50 subtype sensitivity to anthracycline-based chemotherapy, it was shown that the HER2-E subtype received the most benefit, while women with Basal-like tumors had no benefit from this aggressive treatment [31]. This study and the MA.5 trial found that only about two-thirds of clinically Her2+ tumors are classified as HER2-E and about the same percent of triple negatives are classified as Basal-like. Thus, only a subset of the IHC defined groups overlap with PAM50 subtype classification, which may have ramifications for clinical trial findings and predicting therapy benefit.

Receiver Operator Characteristic (ROC) curves are commonly used in medicine to optimize the sensitivity/specificity of an assay depending on the purpose of the test (i.e. screening, monitoring, prognosis, etc.) [32]. In clinical pathology, ROC curves are often used to validate a new methodology against an existing “gold” standard. A major limitation to this approach is that cut-offs are then determined by comparison to an often less than perfect reference. We used an approach for selecting single (*ESR1*, *PGR*, *ERBB2*) and meta-gene (proliferation) cut-offs that was based on the distribution of expression of these markers across the different subtypes. This method showed to be reproducible in an independent test set.

The ROC curves showed high agreement between RT-qPCR and the standard IHC biomarkers. *ESR1* had high sensitivity although the cut-off for ER + status was 10% positive staining nuclei, whereas the new recommendation for determining ER status is 1% [33]. These borderline cases for ER positivity may be better characterized by the overall subtype biology. For *ERBB2*, there was high specificity, which is optimal since confirmatory CISH or FISH would only be performed when it was uncertain if the gene was truly amplified [34]. It has been suggested that the use of single gene RT-qPCR measurement for *ERBB2* is insufficient for determining HER2 positive samples that may benefit from trastuzumab/Herceptin® therapy [35]. Dabbs et al. found that the negative predictive value for determining HER2/*ERBB2* status was high between the HercepTest and the GHI Oncotype Dx qPCR assay (99%); but the concordance for positive HER2/*ERBB2* samples was only 28%. In contrast, we showed that the concordance between HER2 (IHC/CISH) and *ERBB2* (RT-qPCR) is greater than 90% when restricted to the HER2-E subtype.

In order to determine if there was a prognostic difference between the RT-qPCR and IHC we included both methods in a Cox proportional hazards model and showed that gene expression remained significant in the multivariate analysis and replaced IHC. Furthermore, the outcome plots for women with tumors scored positive for ER by IHC but negative for *ESR1* had outcomes similar to women that were ER-/*ESR1*-. Conversely, women with ER- tumors by IHC but positive for *ESR1* had similar outcomes to women with ER+/*ESR1*+ disease. Thus, despite the fact that patients were treated in favor of the IHC diagnosis (i.e. ER + disease was treated with adjuvant tamoxifen) the course of disease was in agreement with the gene expression determination. The better prognosis seen in the ESR1+ but ER- subtype is curious since these patients would not have been given adjuvant endocrine blockade therapy. However, gene expression for ESR1 may be identifying the “true” luminal origin of these tumors which have a better prognosis, regardless of therapy [30]. In addition, the patients included in the test set were locally advanced and received chemotherapy that can cause chemotherapy induced amenorrhea and a reduction in ovarian function [36], which again may benefit the luminal subtype most.

The Normal subtype was developed from reduction mammoplasty “normal” breast tissue and serves as a quality control measure since these cases would be considered to have an insufficient amount of tumor tissue to make a tumor subtype call. Interference studies showed that the introduction of “normal” breast tissue RNA caused a systematic shift in subtype assignment with subtypes switching to Normal, except Luminal B which changed to Luminal A.

None of the assignment switches occurred until the introduction of 50% “normal” breast tissue RNA. The greatest risk of misclassification would come from Luminal B subtypes masquerading as Luminal A tumors because of “normal” tissue contamination [28]; however, these tumors maintain a high proliferation score suggesting they are still a high risk Luminal tumor.

A fifth tumor type that has often been referred to as “Normal-like” has been suggested to be an artifact of having too few tumor cells and a large background of normal breast cells in the sample. Our mixing experiments here support this hypothesis and show that when increasing amounts of “normal” tissue RNA is added to a tumor it switches into the Normal-like group. It is, however, suspected that some tumors now called Normal-like may be put into the recently described Claudin-low classification [37]. The Claudin-low subtype is mostly triple-negative, shares biomarkers in common with normal breast epithelial cells and Basal-like tumors, and may be caused by deficiency in either BRCA1or p53, or both; however there is no clinical indication for Claudin-low, and most are typically classified as Basal-like. There are now many more groups of tumors being identified with transcriptome and copy number variance analyses [38,39]. The overlap between these new groups, existing subtypes, and standard biomarkers already in practice should allow for more personalized treatments and better outcomes in the future.

## Conclusions

Compiling small biomarker panels for the purpose of “intrinsic” subtyping is of limited value in identifying PAM50 based subtypes. Gene expression scoring for *ESR1* and *ERBB2* has good agreement with the corresponding protein biomarkers (ER and HER2) and may have more prognostic power.

## Competing interests

ARUP Laboratories Inc. has a financial interest in the commercial offering of the subject matter. PSB receives research funding from the ARUP Institute for Clinical and Experimental Pathology, although he is not an employee of ARUP. PSB, CMP, and MJE have equity interest in Bioclassifier LLC, which has sublicensed the PAM50 signature from the University of Utah.

## Authors’ contributions

RRLB participated in design of the study, generating data, and drafting the manuscript. ARL participated in recruiting patients, collecting samples and clinical data, and reviewing the manuscript. MTWE participated in design of the study, bioinformatics and statistical analysis, and drafting the manuscript. AP participated in bioinformatics and statistical analysis. BM participated in recruiting patients, collecting samples and clinical data, and reviewing the manuscript. LR participated in design of the study. PM participated in design of the study. MRB participated in recruiting patients, collecting samples and clinical data, and reviewing the manuscript. DA participated in design of the study. BL participated in manufacturing of the PCR plates. IA performed IHQ/CISH on GEICAM/9906 samples and participated in reviewing the manuscript. TD participated in manufacturing of the PCR plates. DW participated in manufacturing of the PCR plates. MAS participated in recruiting patients, collecting samples and clinical data, and reviewing the manuscript. LB participated in manufacturing of the PCR plates. KMB participated in statistical analysis. EA participated in recruiting patients, collecting samples and clinical data, and reviewing the manuscript. LP participated in statistical analysis. CAD participated in sample preparation and organization. IA participated in recruiting patients, collecting samples and clinical data, and reviewing the manuscript. CF participated in robotics design. IJS participated in data generation and reviewing the manuscript. JP participated in recruiting patients, collecting samples and clinical data, and reviewing the manuscript. AA participated in recruiting patients, collecting samples and clinical data, and reviewing the manuscript. EC participated in design of the study and reviewing the manuscript. RC participated in design of the study, managing the collection of samples and central laboratory activity, and reviewing the manuscript. MJE participated in reviewing the manuscript. TON participated in reviewing the manuscript. CMP participated in collecting samples and clinical data, and reviewing the manuscript. MA participated in the conceiving and design of the study. PSB participated in conceiving and design of the study, and drafting the manuscript. MM participated in design of the study, recruiting patients, collecting samples and clinical data, and reviewing the manuscript. All authors read and approved the final manuscript.

## Pre-publication history

The pre-publication history for this paper can be accessed here:

http://www.biomedcentral.com/1755-8794/5/44/prepub

## Supplementary Material

Additional file 1 **Clinical-pathological information associated with training set subtypes.** Clinical-pathological information associated with the 171 samples included in the training set. (XLSX 23 kb) Click here for file

Additional file 2 **Clinical-pathological information and PAM50 data associated with GEICAM/9906 test set.** Clinical-pathological information and PAM50 RT-qPCR results associated with the 814 samples included in the GEICAM9906 test set. (XLS 1106 kb) Click here for file

Additional file 3 **Additional materials and methods.** Methods for plate manufacturing, PCR, calculation of log-expression ratios, PCR-efficiency, limits of detection, and limits of quantification are described (Additional files 8 and 9). (DOCX 213 kb) Click here for file

Additional file 4 **10-fold cross validation of training set.** Each gene was measured in triplicate per RT-qPCR run on the Roche LC480 and 2 runs were performed for each of the 17 dilutions. The prototype samples identified by SigClust were split into 10 groups and nine of the 10 groups were used to calculate new centroids for each of the 5 possible subtype assignments. (XLSX 12 kb) Click here for file

Additional file 5 **Interference in subtype call and single/meta-gene scores from normal contamination.** Interference by normal cell contamination of subtype call and single and meta-gene classes is shown. The changes in subtype classification occurred in a systematic fashion with all subtypes switching to a classification of Normal/Insufficient, with the exception of Luminal B, which switched to Luminal A. (XLSX 10 kb) Click here for file

Additional file 6 **Single and meta-gene cutoffs.** Data from single and meta-gene expression score over the GEICAM 9906 samples are plotted on the 1–10 scale. The cut-points between high, intermediate, and low classes were individually derived from the training set. Samples are color-coded according to immunohistochemistry positivity (red) or negativity (blue), except in the case of the training set proliferation score where samples are colored by high, intermediate or low proliferation class. Luminal score samples are colored as being ER+/PR+, ER + or PR + (positive, red), and ER-/PR- (negative, blue). (PDF 1524 kb) Click here for file

Additional file 7 **Hierarchical clustering for GEICAM 9906.** A comparison of unsupervised hierarchical clustering with supervised subtype assignment and single marker scores for GEICAM 9906. (PDF 1616 kb) Click here for file

Additional file 8 **PCR Efficiency, limits of detection, and limits of quantification.** Supplemental table listing the efficiency of PCR, limits of detection, and limits of quantification for the 50 classifier and 5 housekeeper genes of the PAM50. Data are from 34 runs across 17 dilutions from a mixture of 8 breast cancer cell lines. Click here for file

Additional file 9 **Reproducibility of PAM50 gene measurements.**Within plate, within plate batch and across plate batch coefficient of variation for the 50 classifier and 5 housekeeper genes of the PAM50 were calculated using cell lines and a tumor samples. Click here for file
