# Supplementary material for: PAM50 Breast Cancer Subtyping by RT-qPCR and Concordance with Standard Clinical Molecular Markers
Source: BMC Med Genomics. 2012 Oct 4;5:44. doi: 10.1186/1755-8794-5-44 (PMC3487945; doi:10.1186/1755-8794-5-44)
Supplement: Additional file 3 — Additional materials and methods. Methods for plate manufacturing, PCR, calculation of log-expression ratios, PCR-efficiency, limits of detection, and limits of quantification are described (Additional files 8 and 9). (DOCX 213 kb) [file 1755-8794-5-44-S3.docx]

**PAM50 RT-qPCR Plate Design and Manufacturing**

Each 384-well PCR plate (Roche Applied Sciences, Indianapolis, IN, www.roche-applied-science.com) had six wells designated for each of the 55 genes: 1 no template control (negative control), reference cDNA (positive control), and 3 experimental sample wells. Each gene had forward and reverse primers (2 picomoles/well) pipetted into all 6 wells using an Innovadyne Screenmaker liquid handler (Innovadyne Technologies, Rohnert Park, CA, www.innovadyne.com). Primers were HPLC purified and required to be at least 85% full length. Reference cDNA (30ng/well) was composed of a mixture of breast cancer cell lines T47D and MDA-MB-468 in a 2:1 ratio respectively (ATCC, Manassas, VA, www.atcc.org). Primers and cell-line reference were dried-down by centrifugation in a 9mbar vacuum at 60°C for 70min using a Genevac Quattro centrifuge (Genevac, Gardiner, NY, www.genevac.com). The plates were sealed and stored at -20°C until real-time PCR was performed.

**RNA Extraction, Reverse Transcription, and PCR**

RNA was extracted from formalin-fixed, paraffin-embedded (FFPE) tissue blocks starting with 2x10μm full-face sections or 2x1.5mm tumor-directed punches. Samples were deparaffinized using Citrus Clearing Solvent (Richard-Allen Scientific, Kalamazoo, MI, www.thermofisher.com) followed by dehydration in absolute ethanol. RNA extraction was completed on a Biomek NX Laboratory Automation Workstation (Beckman Coulter, Beverly, MA, www.beckmancoulter.com) using the AgenCourt FormaPure Kit (Beckman Coulter, Beverly, MA, www.beckmancoulter.com) according to the manufacturer’s instructions and included a DNase I step. RNA quantification was performed on a Paradigm Detection Platform (Beckman Coulter, Beverly, MA, www.beckmancoulter.com) using the Quant-iT RiboGreen Assay Kit (Invitrogen, Carlsbad, CA, www.invitrogen.com). Synthesis of cDNA was performed on the Biomek FX Laboratory Automation Workstation (Beckman Coulter, Beverly, MA, www.beckmancoulter.com) using 600ng of RNA, uracil containing dNTPs (Invitrogen, Carlsbad, CA, www.invitrogen.com), random primers (Invitrogen, Carlsbad, CA, www.invitrogen.com), gene-specific, downstream PCR primers (Idaho Technology, Salt Lake City, UT, www.idahotech.com), and SuperScript III Reverse Transcriptase (Invitrogen, Carlsbad, CA, www.invitrogen.com).

Each 5uL reaction contained 1X LightCycler 480 SYBR Green I Master Mix (Roche Applied Sciences, Indianapolis, IN, www.roche-applied-science.com) and 1.67ng cDNA was added to the experimental sample wells. Sample cDNA was incubated with LightCycler Uracil-DNA Glycosylase (Roche Applied Sciences, Indianapolis, IN, www.roche-applied-science.com) at 40°C for 10min and inactivated at 95°C for 10min prior to PCR. Real-time PCR was performed on the LightCycler (LC) 480 (Roche Applied Sciences, Indianapolis, IN, www.roche-applied-science.com) as follows: 45 cycles of 95°C for 4sec, 58°C for 6sec and 72°C for 6sec. To assure target specificity, PCR was followed by a melting curve analysis: 95°C for 15sec, 65°C for 1min followed by raising the temperature to 99°C while taking 10 fluorescence acquisitions/°C.

**Quality Control and Relative Quantification**

The raw data from the LC480 run (.ixo file) was exported to the PAM50 software, which verifies the manufacturing batch of the PCR plate and the experiment name. After the .ixo file is authenticated, the run is evaluated for overall quality. The crossing point (C_P_) for each reference calibrator must be within the pre-determined range of the specific batch, the melting temperature (T_M_) of each sample replicate must be the same as the calibrator for target specificity, there should be minimal primer-dimer formation (non-specific peak height less than three-quarters the specific peak height), and no amplification in the negative control. A gene fails if the gene’s positive control fails or the negative control fails and a run fails if more than 2 classifier genes fail or if a single housekeeper gene fails.

**Quality Control and Relative Quantification**

Relative quantification was done by comparing the C_p_ of each sample well for each gene to the mean of its calibrator, known as a calibrated ratio. An external efficiency curve was used to calculate the calibrated ratio between the reference calibrator and experimental samples. The median calibrated ratio was used for each gene. A log-expression ratio was calculated using the median calibrated ratio of the classifier gene and then subtracted by the geometric average of the 5 housekeeper genes (Equation 1).

Equation 1. Log-Expression Ratio


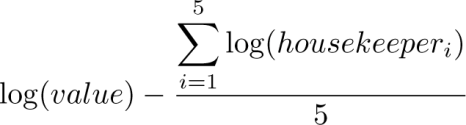


To compensate for variation between plate batches, an adjustment is made to the final log expression ratio for each individual gene in a given batch. The size of the adjustment is determined by running 10 quality-control plates in which the cDNA calibrator is run as a substitute for the tumor sample. When running the reference sample as a tumor sample, the log-expression ratio for each of the 50 classifier genes should theoretically be 0. The adjustment value for a single gene in a given batch is calculated by taking the difference between the median of the actual log expression ratios across all quality control plates in the batch and the theoretical value of 0. The calculation is repeated for each of the 50 classifier genes.

***PCR Efficiency***

Amplification efficiencies for each of the 55 genes were estimated using Equation 2, where slope is estimated from the simple linear regression of C_P_ measurements vs. log_2_ RNA concentration. A mean C_P_ value was calculated from the six measurements for use in the simple linear regression analysis. Reactions for which the T_M_ was non-specific were excluded from the analysis.

Equation 2.

**
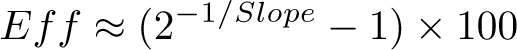
**

***Limits of Detection and Limits of Quantification***

Limits of Detection (LOD) is an estimate of the minimum RNA concentration that can be detected by the assay. A 95% confidence interval was used for calculating the LOD using Equation 3, where μ_cp_ is the mean C_P_ of all negative wells for the gene of interest, σ_cp_ is the standard deviation of the negative wells for the gene of interest and 1.645 is the z-score for a one-sided 95% confidence interval. Only the lower values of the 95% confidence interval were used.

Equation 3.


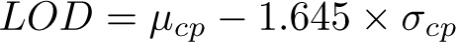


Limits of Quantification (LOQ) is an estimate of the minimum RNA concentration that can be quantified with a high degree of confidence. LOQs were calculated for each gene by using the inverse prediction of C_P_ values from the upper 95% confidence interval of the best fitting linear line for the data. Initially, the data was analyzed to determine the best fitting polynomial model between linear, quadratic and cubic models. The SAS PROC MIXED procedure (SAS version 9.2) was used to calculate the width of the 95% confidence interval (Equation 4) where a p-value was used to select the best fitting model. The values of the upper 95% confidence interval were used to predict the LOQ by solving Equation 4 for x, and plugging x into Equation 5 to estimate the maximum C_P_ that can be detected with a high degree of confidence.

Equation 4.


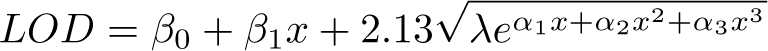


Equation 5.


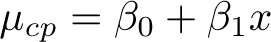


**PAM50 RT-qPCR Single Gene Analytical Performance Characteristics**

Each of the PAM50 genes and 5 control genes were individually analyzed for PCR amplification efficiencies, limits of detection (LOD) and limits of quantification (LOQ). The mean PCR efficiency was 1.91 (SD 0.28, range 1.72 – 3.9), the mean LOD by C_P_ was 39.93 (SD 0.31, C_P_ range 38.36 – 40), and the mean LOQ by C_P_ was 36.30 (SD 0.71, C_P_ range 34.83 – 38.93). Values for each gene are provided in Additional File 8. The variation in ∆C_P_ for each gene within plate, within batch, and across batches is provided in Additional File 9.
