# Supplementary material for: PAM50 Breast Cancer Subtyping by RT-qPCR and Concordance with Standard Clinical Molecular Markers
Source: BMC Med Genomics. 2012 Oct 4;5:44. doi: 10.1186/1755-8794-5-44 (PMC3487945; doi:10.1186/1755-8794-5-44)
Supplement: Additional file 6 — Single and meta-gene cutoffs. Data from single and meta-gene expression score over the GEICAM 9906 samples are plotted on the 1–10 scale. The cut-points between high, intermediate, and low classes were individually derived from the training set. Samples are color-coded according to immunohistochemistry positivity (red) or negativity (blue), except in the case of the training set proliferation score where samples are colored by high, intermediate or low proliferation class. Luminal score samples are colored as being ER+/PR+, ER + or PR + (positive, red), and ER-/PR- (negative, blue). (PDF 1524 kb) [file 1755-8794-5-44-S6.pdf]

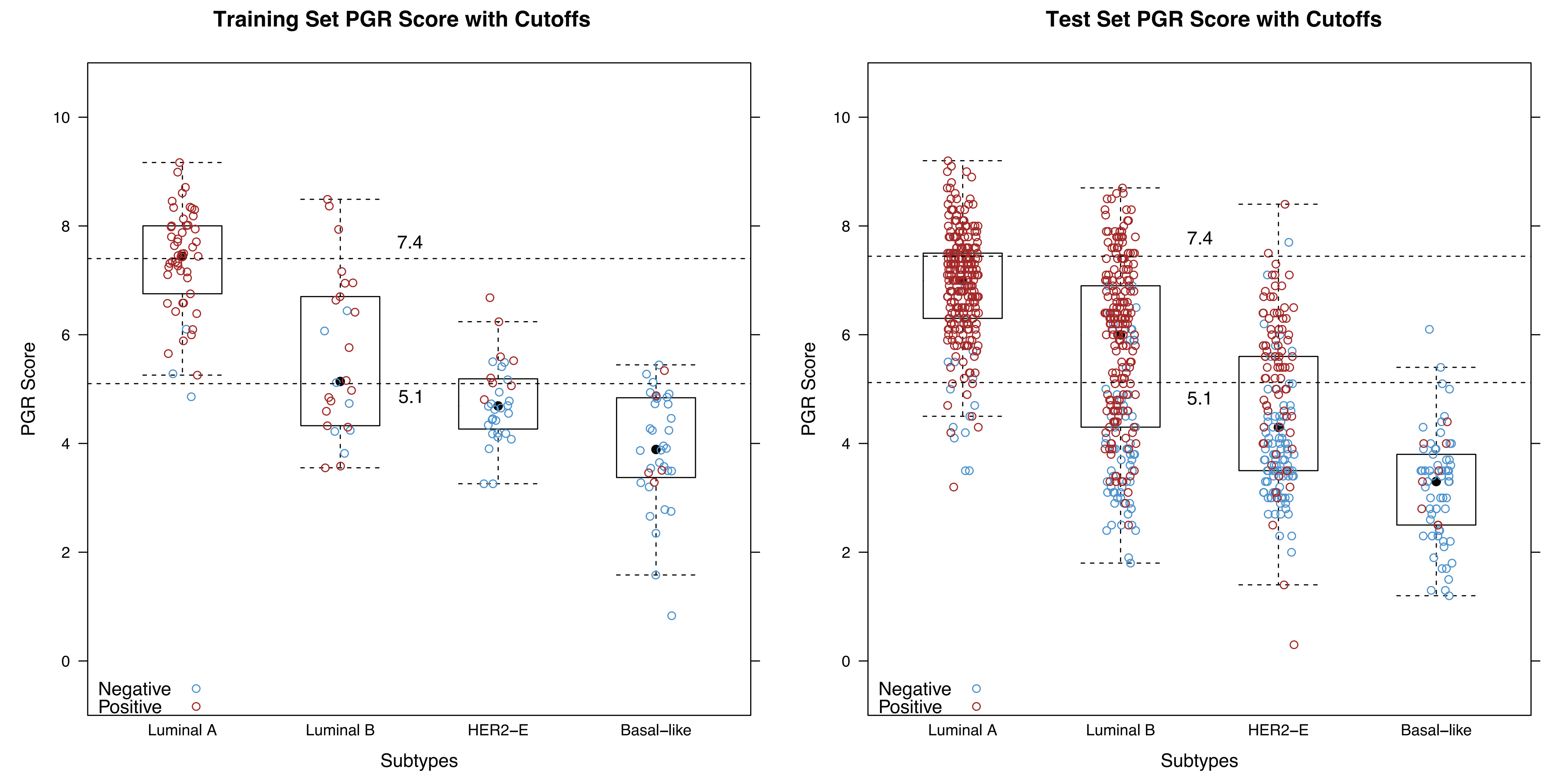
Training Set PGR Score with Cutoffs
Test Set PGR Score with Cutoffs
PGR Score
10
8
6
4
2
0
7.4
5.1
Negative
Positive
Luminal A
Luminal B
HER2–E
Basal–like
Subtypes

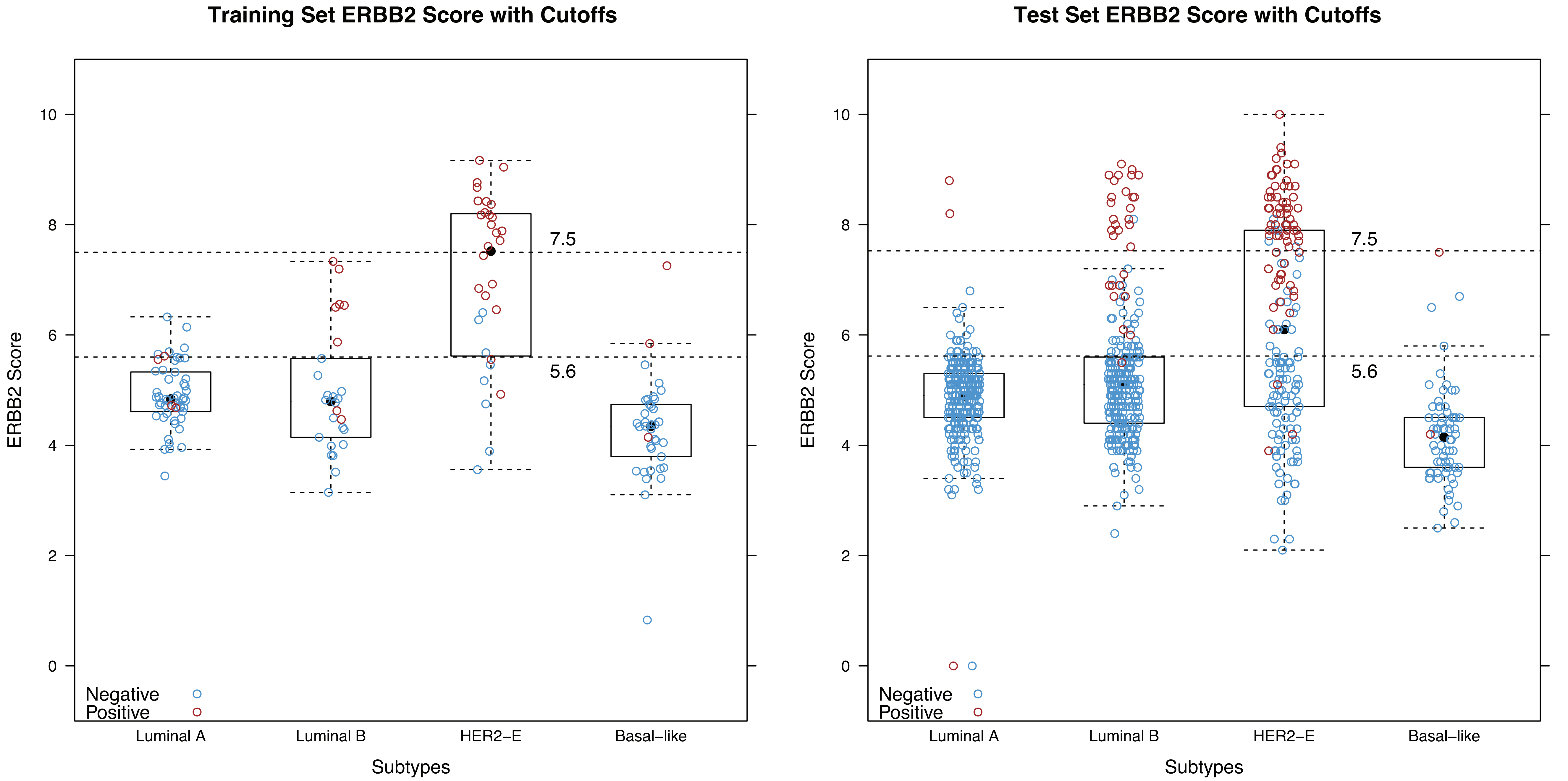
Training Set ERBB2 Score with Cutoffs
Test Set ERBB2 Score with Cutoffs
ERBB2 Score
Subtypes
Luminal A
Luminal B
HER2−E
Basal−like
7.5
5.6
0
2
4
6
8
10
Negative
Positive

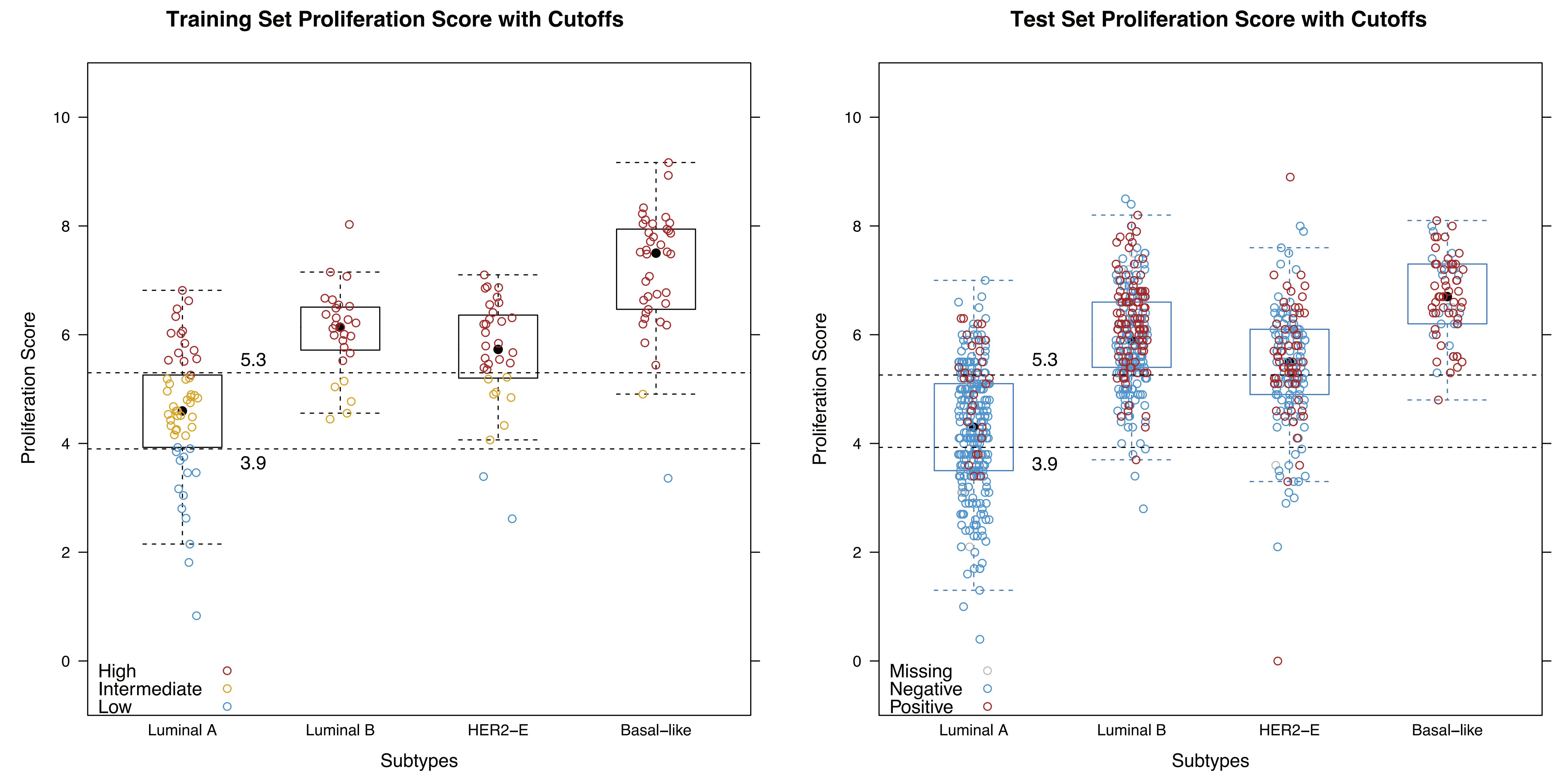

Training Set Proliferation Score with Cutoffs
Test Set Proliferation Score with Cutoffs
Proliferation Score
10
8
6
4
2
0
5.3
3.9
High
Intermediate
Low
Missing
Negative
Positive
Luminal A
Luminal B
HER2−E
Basal−like
Subtypes
