# Supplementary figures and images for: PAM50 Breast Cancer Subtyping by RT-qPCR and Concordance with Standard Clinical Molecular Markers
Source: BMC Med Genomics. 2012 Oct 4;5:44. doi: 10.1186/1755-8794-5-44 (PMC3487945; doi:10.1186/1755-8794-5-44)

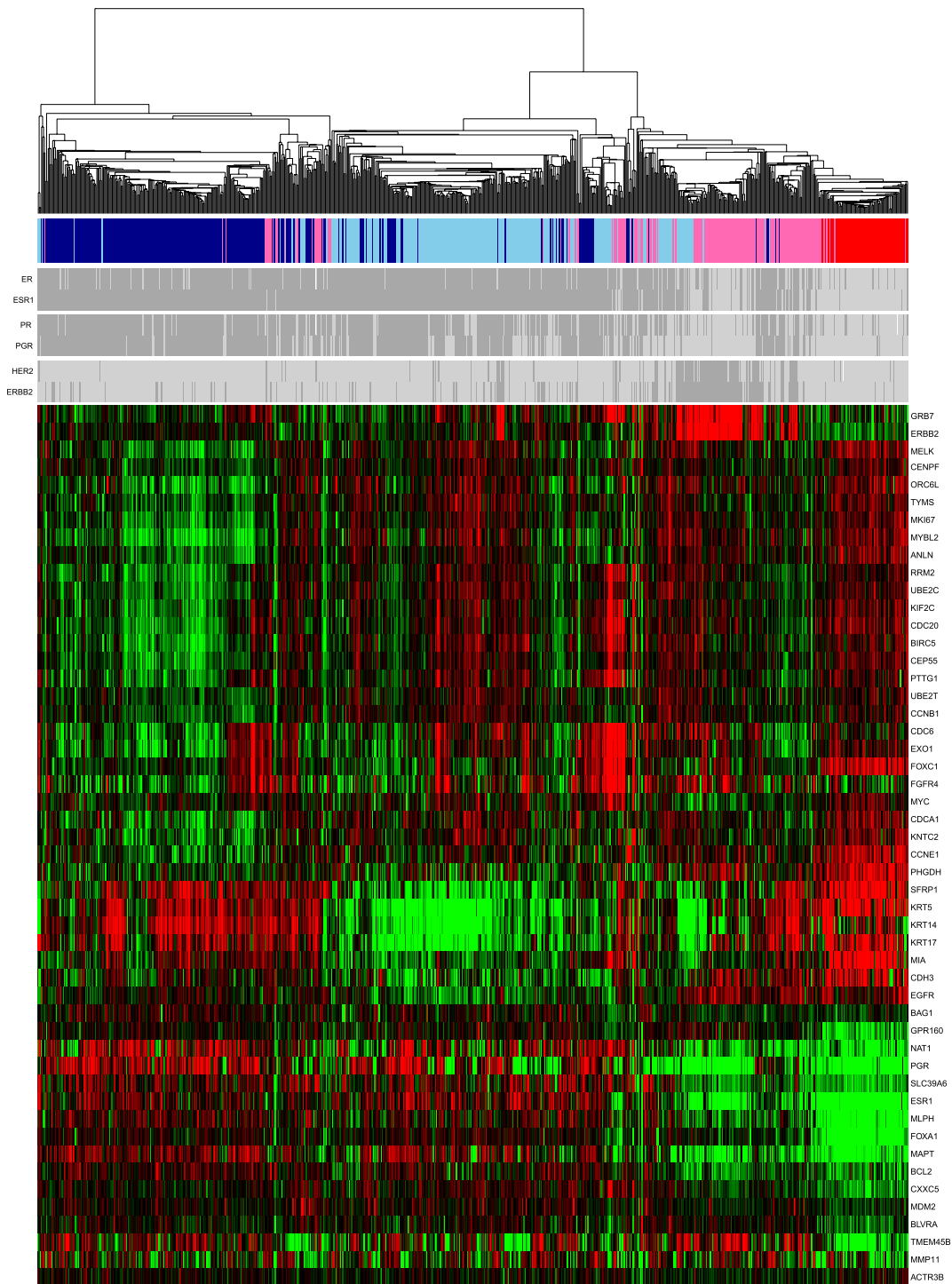

Supplement: Additional file 7 — Hierarchical clustering for GEICAM 9906. A comparison of unsupervised hierarchical clustering with supervised subtype assignment and single marker scores for GEICAM 9906. (PDF 1616 kb) [file 1755-8794-5-44-S7.pdf]
